# Hispanic Immigrants and Suicide: Overcoming Data Challenges in an Anti-immigrant Climate

**DOI:** 10.1016/j.focus.2022.100038

**Published:** 2022-10-01

**Authors:** Evan V. Goldstein, Fernando A. Wilson

**Affiliations:** 1 Department of Population Health Sciences, Spencer Fox Eccles School of Medicine, University of Utah, Salt Lake City, Utah; 2 Department of Economics, University of Utah, Salt Lake City, Utah; 3 Matheson Center for Health Care Studies, University of Utah, Salt Lake City, Utah

**Keywords:** Suicide, immigrant health, health disparity, health policy

Anti-immigrant political rhetoric has increased in the U.S. since 2015,^1^^,^^2^ inciting new concerns about the health and well-being of Hispanic and Latino immigrants. Of particular worry is the current state of untreated mental illness and suicide risk in Hispanic immigrant communities. From traditional media outlets, there have been reports of despair among immigrant youth and tragic stories of deportation fears leading to suicide contagion haunting entire Hispanic communities.^3^ However, alarmingly little is understood about the current burden of suicidal ideation, attempts, and deaths among authorized or unauthorized Hispanic immigrants living in the U.S. in relation to the rise in anti-immigrant sentiment.^4^

Although suicide death is more common among non-Hispanic White persons, we know from violent death surveillance data that Hispanic suicide rates rose by 26.6% from 2015 to 2020, compared with an increase of 0.13% in non-Hispanic suicide rates over the same period.^5^ Older estimates from the Collaborative Psychiatric Epidemiology Surveys suggest that about 7.8% and 2.9% of 566 Mexican-born migrants living across the U.S. experienced suicidal ideation and attempted suicide, respectively, from 2001 to 2003.^6^ A more recent survey (2018–2019) of adolescents from Michigan and Virginia showed an association between being born outside the U.S. and suicidal ideation; however, most of the study's participants were from the Middle East and North Africa.^7^

We do not know how much of the rise in Hispanic suicide rates is attributable to immigrant deaths. We also do not understand how suicide and suicidal ideation changed for different Hispanic immigrant populations since 2015, coinciding with a rise in anti-immigrant political sentiment and policymaking. Without action to either improve public health surveillance or leverage existing data sources with immigration status information to better understand the burden of suicide death and suicide risks among Hispanic immigrants, we may fail to prevent unnecessary loss of life.

## ANTI-IMMIGRANT CLIMATE IN THE U.S

Anti-immigrant attitudes are not a new phenomenon in the U.S.,^1^ and anti-immigrant rhetoric and discriminatory policies have evolved slowly since the early 1900s.^8^^,^^9^ State-level policies such as California's Proposition 187 (1994) and federal policies such as the Personal Responsibility and Work Opportunity Reconciliation Act (1996) and Illegal Immigration Reform and Immigrant Responsibility Act (1996) stoked immigration fears and impacted the social identity and health of unauthorized and authorized Latino populations.^10^^,^^11^ Similarly, the recent rise in anti-immigrant political rhetoric likely exacerbated feelings of social isolation and mental ill health experienced by many Hispanic immigrants, whereas public policy decisions made by the Trump administration likely dissuaded many immigrants from accessing critical public health resources.

A key example of these policies was the administration's highly publicized public charge rule changes, which built upon the public charge standard developed through the Illegal Immigration Reform and Immigrant Responsibility Act.^12^ These new regulations sought to allow federal officials to consider noncash government assistance as grounds for rejection during the immigration process, including insurance subsidies and Children's Health Insurance Program/Medicaid coverage for eligible children in immigrant families.^13^ Although the 2019 rule changes have since been halted, these and other policies likely compounded the harmful effects of the rise in anti-immigrant rhetoric. By 2019, more than half (52%) of foreign-born Hispanics had serious concerns about their place in the country with Donald Trump as president, and nearly 4 in 10 Hispanics reported experiencing discrimination in 2019.^14^ Unlike some public policies, the consequences of fear and psychological trauma are not easily reversed.

## IMMIGRATION POLICY AND SUICIDE RISK

Unfavorable immigration policy has been linked to greater perceived health problems among adults and children.^15^^,^^16^ Hispanic suicide risk, in particular, varies across nationality and between U.S.- and foreign-born populations.^4^ Studies predating the latest acrimonious immigration climate suggest that foreign-born Hispanics (7.6%) in the U.S. tend to have lower lifetime rates of suicidality than U.S.-born natives (15.5%)^17^ as well as lower rates of suicide death.^4^ Yet, older studies also show that acculturation stresses and exposure to discrimination can increase the risk of suicidal ideation and suicide attempts among Hispanic immigrants.^18^ In addition to suicide-specific phenotypes, poor mental health outcomes have been shown to be higher among Latinos in states with more exclusionary immigration policy climates,^19^ whereas increased arrest rates after restrictive immigration policies have been associated with worsening mental health among Hispanics.^20^

Despite these risks, deportation fears and the chilling effects of anti-immigrant attitudes and policies likely discouraged enrollment into health insurance programs (e.g., local coverage programs for unauthorized immigrants)^13^^,^^21^ and deterred the use of needed mental health services. In the 1990s, the enactment of California's Proposition 187 was followed by a 26% decrease in outpatient mental healthcare visits by younger Hispanic patients at facilities serving large Hispanic immigrant populations in San Francisco County.^11^

Avoiding care while facing discrimination or psychological trauma can be problematic for suicide prevention. Discrimination is a risk factor for mental health problems among Hispanic persons.^22^ Mental illness is also strongly linked to suicidality. Unfortunately, many suicide decedents in the U.S. do not engage the health system before taking their lives. Even among those seeking or receiving care, mental illness often goes undiagnosed before suicide death.^23^ This is particularly concerning given evidence suggesting less favorable attitudes toward help seeking and a lower likelihood of using mental healthcare among Hispanics than among the general population.^24^^,^^25^

## A CALL TO ACTION: BETTER DATA AND LEVERAGING EXISTING DATA

In response, public health researchers have an opportunity to increase their understanding of the burden of suicide among Hispanic immigrant populations in relation to the rise of anti-immigrant sentiment. This remains a challenging task when national mental health and suicide statistics do not take immigration status into account, when commonly available data sources restrict access to information about visa status, and when data from the Centers for Disease Control and Prevention do not distinguish between immigrants and U.S.-born Hispanics.

Many researchers have attempted to account for immigration status in their studies―cutting across multiple disciplines―although these attempts have been discordant, resulting in different definitions and experiences of unauthorized legal status.^26^ Moreover, given the sensitive nature of this topic, efforts to systematically measure legal status may be stigmatizing. Collecting citizenship status information from unauthorized immigrants could be particularly challenging because of deportation-related fears, especially amid anti-immigrant public attitudes. Nevertheless, equipped with better surveillance data, the scientific community can play a crucial role in analyzing and communicating new knowledge about suicide risks for different Hispanic immigrant populations.

Specific actions can be taken while being mindful of stigma-related implementation concerns. First, rather than actively seeking information from Hispanic immigrants, we can prioritize efforts to report deceased immigrants’ citizenship status on either the U.S. Standard Certificate of Death or equivalent state-level forms. Specifically, citizenship status, available by contacting Citizenship and Immigration Services, could feasibly be collected by the medical certifier or funeral director when collecting demographic data required for the death certificate. This information would then be reported into state violent death report systems, which already aggregate deidentified information on country of birth and other sensitive characteristics for suicide decedents.

Second, additional initiatives could include collecting citizenship information for immigrants participating in large health surveys. Previous studies suggest that unauthorized immigrants are interested in and willing to discuss legal status in research. To ease the impact of the deportation-related concerns on unauthorized immigrant participation, surveyors should build rapport before presenting legal status questions^26^ and ensure that survey responses will be deidentified for public use. Even standardizing the collection of country of birth information would be a step forward, allowing researchers to impute immigration status using machine learning or other methods. Alternatively, the public health community could agree on standard procedures for imputing decedents’ citizenship status in violent death reporting system data using existing country of birth, social security number, race/ethnicity, and other variables predictive of immigration status.

Third, the research community can use existing data in creative ways to better understand suicide-related risks experienced by Hispanic immigrants. Data sources collecting citizenship or country of birth information, such as the National Health Interview Survey or Hispanic Community Health/Study of Latinos, respectively, can be used to estimate the relationships between immigration status and important suicide risk factors for Hispanic populations since 2015. Existing data sources can also give us guidance on how to estimate the impact of recent immigration policies on suicide risk factors such as mental health outcomes.^19^ For example, one recent study used data from the Los Angeles Family and Neighborhood Survey and machine learning methods to predict the authorization status of Medical Expenditure Panel Survey respondents, which lacks citizenship information.^27^ These actions would help researchers to quantify the population-level burden of Hispanic immigrant suicide in the U.S. and characterize important social and health-related circumstances preceding suicide for both authorized and unauthorized immigrants after the rise in anti-immigrant sentiment.

## CONCLUSIONS

We do not fully understand the burden of suicide or recent changes in suicidal ideation among Hispanic immigrant populations in the U.S. However, we hypothesize that increasingly acrimonious immigration attitudes and policies likely worsened suicide-related outcomes for many Hispanic immigrants, authorized and unauthorized. The coronavirus disease 2019 (COVID-19) pandemic is likely intensifying suicide-related risks, given the harmful impacts the pandemic has imposed on Hispanic communities.^28^ With better data and creatively using existing data, the scientific community can play a crucial role in understanding the burden of suicide and identifying critical suicide risk factors among Hispanic immigrants amid the current rise in anti-immigrant attitudes.
